# Visualization of Mouse Neuronal Ganglia Infected by Herpes Simplex Virus 1 (HSV-1) Using Multimodal Non-Linear Optical Microscopy

**DOI:** 10.1371/journal.pone.0105103

**Published:** 2014-08-18

**Authors:** Pierre-Alexandre Rochette, Mathieu Laliberté, Antony Bertrand-Grenier, Marie-Andrée Houle, Marie-Claire Blache, François Légaré, Angela Pearson

**Affiliations:** 1 INRS – Institut Armand-Frappier, Laval, Québec, Canada; 2 INRS – Centre Énergie Matériaux Télécommunications, Varennes, Québec, Canada; UC Irvine Medical Center, United States of America

## Abstract

Herpes simplex virus 1 (HSV-1) is a neurotropic virus that causes skin lesions and goes on to enter a latent state in neurons of the trigeminal ganglia. Following stress, the virus may reactivate from latency leading to recurrent lesions. The *in situ* study of neuronal infections by HSV-1 is critical to understanding the mechanisms involved in the biology of this virus and how it causes disease; however, this normally requires fixation and sectioning of the target tissues followed by treatment with contrast agents to visualize key structures, which can lead to artifacts. To further our ability to study HSV-1 neuropathogenesis, we have generated a recombinant virus expressing a second generation red fluorescent protein (mCherry), which behaves like the parental virus *in vivo*. By optimizing the application of a multimodal non-linear optical microscopy platform, we have successfully visualized in unsectioned trigeminal ganglia of mice both infected cells by two-photon fluorescence microscopy, and myelinated axons of uninfected surrounding cells by coherent anti-Stokes Raman scattering (CARS) microscopy. These results represent the first report of CARS microscopy being combined with 2-photon fluorescence microscopy to visualize virus-infected cells deep within unsectioned explanted tissue, and demonstrate the application of multimodal non-linear optical microscopy for high spatial resolution biological imaging of tissues without the use of stains or fixatives.

## Introduction

Immunohistochemical approaches to study viral pathogenesis can be highly informative but they require harsh chemical fixation as well as thin sectioning of tissue because of the poor depth penetration of traditional light microscopy. Both of these treatments have been demonstrated to affect tissue morphology [1], and furthermore, the three-dimensional structure of the tissue is necessarily lost. Bioluminescence strategies are useful for *in vivo* studies; however, the resolution is relatively poor. In the past years, the use of fluorescent proteins to study viral infections has grown [2], [3], [4]. One advantage of this approach over immunohistochemistry is that there are no concerns of non-specific staining due to cross-reactivity of antibodies with different antigens. But traditional confocal microscopy does not permit imaging deep within tissue, therefore it typically necessitates sectioning of the tissue. In contrast, two-photon microscopy allows imaging deep (1 mm) in tissue such that intrinsically fluorescent proteins expressed within tissue can be imaged at high resolution in a three dimensional tissular context. Despite these advantages, two-photon fluorescence imaging cannot match traditional histochemistry with regards to distinguishing different tissue components. In addition to two-photon microscopy, other microscopy techniques, which are label-free, are used by the biomedical community to image tissues including second harmonic generation [5], [6], [7], [8] and coherent anti-Stokes Raman scattering (CARS) [9], [10], [11]. CARS microscopy provides an imaging contrast based on vibrational spectroscopy and is extremely powerful to image structures rich in CH_2_ symmetric stretching modes, a chemical group which is abundant in lipids [12], [13], [14], [15], [16], [17]. This type of microscopy has already been exploited to visualize lipid droplets induced by the Hepatitis C virus (HCV) in cell culture [18]. In whole tissue mount, multiphoton fluorescence microscopy can be used to identify different structures by their autofluorescence, and it affords great sectioning capability [19], [20]; however, autofluorescent properties of the tissue under study can be problematic if the emission spectra overlap with that of the fluorescent protein used for tracking of cells [21]. Optimization of the experimental conditions for each imaging modality, as well as their compatibility, is crucial if one is to combine their respective strengths to achieve multimodal high spatial resolution imaging deep in tissue.

Herpes simplex virus 1 (HSV-1) is a neurotropic virus. The double-strand DNA genome is contained within an icosahedral capsid surrounded by a protein rich tegument, and finally a bilipid envelope [22], [23]. During a primary infection, the virus first replicates in epithelial cells of the mucous membrane leading to skin lesions commonly referred to as cold sores [24], [25]. Next, the virus infects the endings of sensory neurons innervating the mucosa and spreads to the trigeminal ganglia (TG). The virus can replicate within neurons of the TG, though ultimately, viral gene expression in the neuronal ganglia will diminish, and a life-long latent infection will be established [26], [27], [28], [29]. Under conditions of stress, the virus can exit latency and re-enter a productive cycle of viral replication leading to recurrent infections. While infections in healthy individuals are usually benign, ocular keratitis and viral encephalitis can occur. In immune-compromised hosts and in neonates, infections can be more severe and may lead to disseminated disease [30], [31]. The murine ocular infection model for HSV-1 recapitulates many of the stages of a typical infection including acute replication in the mucosa, acute replication in neurons of the TG, and the establishment of a latent infection [32], [33]. *Ex vivo* reactivation upon post-mortem harvesting of TG is also observed, and virus reactivation *in vivo* (to varying degrees) can be induced through different means [34], [35], [36], [37].

The goal of this study was to achieve multimodal high spatial resolution imaging deep within an HSV-1-infected TG using signals intrinsically generated by, and generated within the tissue. TG contain many myelinated neurons of various sizes, whose myelin sheathes can generate a strong and specific CARS signal [12], [14], [17] by virtue of their richness in symmetric CH_2_ vibrational modes. The neuron soma generates very little CARS signal, but is strongly autofluorescent and emits in the green spectrum close to that of the commonly used green fluorescent protein [21]. Infection of cells can be detected using a virus expressing a fluorescent reporter gene. To date, the combination of CARS and multiphoton fluorescence microscopy in the context of a viral infection has been limited to the analysis of single monolayers of cells in culture [18], [38]. Herein, we describe for the first time the combined use of CARS microscopy and two-photon fluorescence microscopy to visualize infected cells and components of the surrounding tissue in an unfixed and unsectioned virus-infected tissue.

## Results

### Production of vUs7-8mCherry

**Figure 1:**
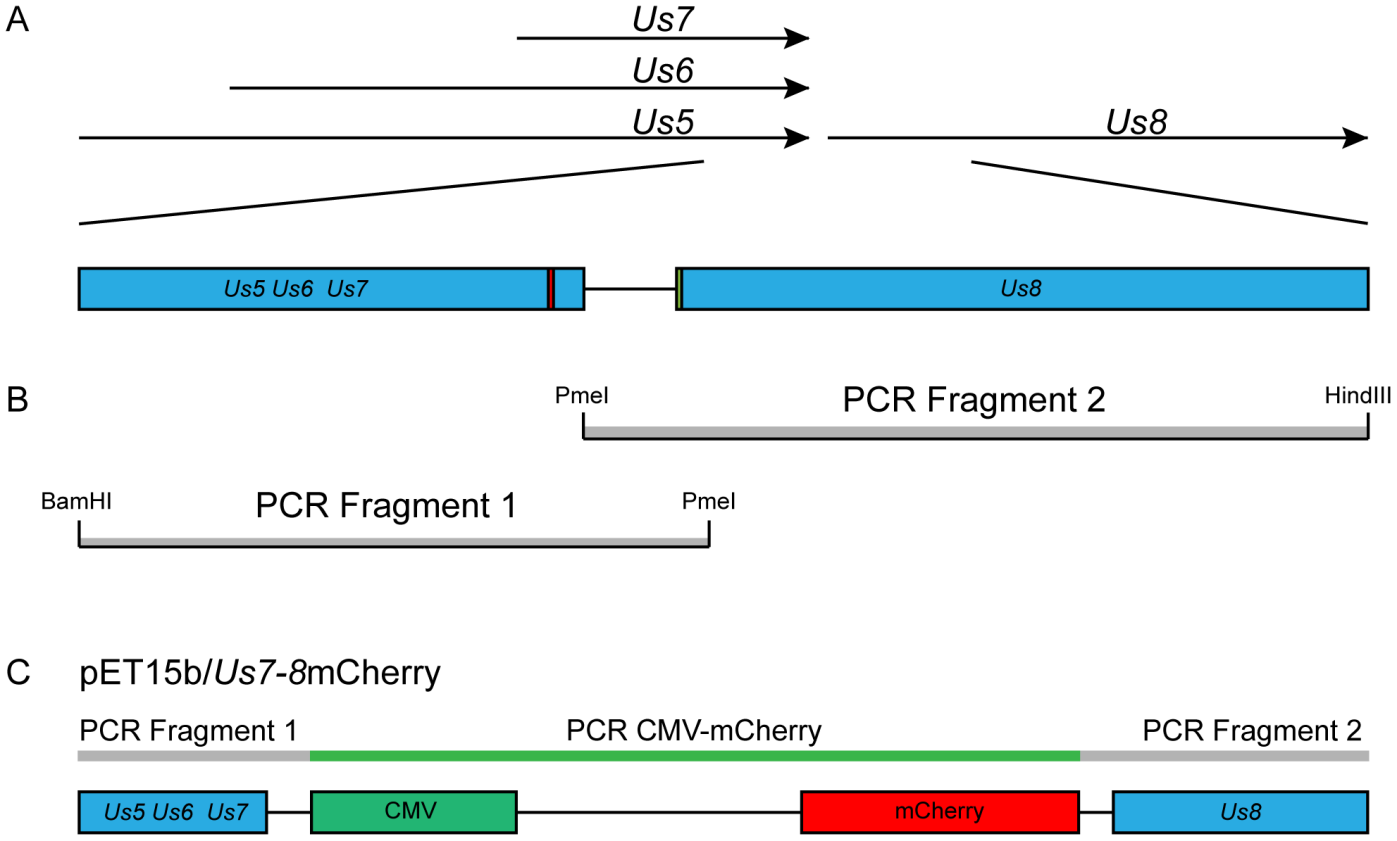
Production of a recombinant strain of HSV-1 expressing a red fluorescent protein. The region between the viral genes *Us7* and *Us8* was chosen as the site of insertion of an expression cassette for the red fluorescent protein mCherry. (A) The arrows indicate the open reading frames (ORF) of viral genes present in the locus. The area of interest modified by PCR involves the end of the transcribed region of the viral genes *Us5*, *Us6*, and *Us7*, as well as the beginning of the transcribed region of the viral gene *Us8*, both indicated by blue boxes. The red box indicates the location of the Poly A signal shared by *Us5*, *Us6*, and *Us7*. The green box indicates the location of the *Us8* start codon. The line between the *Us5-7* and the *Us8* boxes indicates the intergenic site flanked by both transcribed regions. (B) The intergenic region was duplicated in tandem following amplification by two PCR reactions. The grey boxes represent each of the fragments generated by PCR. The restriction sites embedded within the primers used for the PCR are indicated at the ends of each fragments. (C) The transfer vector was produced following the insertion of a mCherry expression cassette between the duplicated intergenic region of *Us7* and *Us8*. The thin grey rectangles represent the PCR fragments. The thin green rectangle represents the mCherry expression cassette. The blue boxes represent the viral ORFs of genes *Us5*, *Us6*, *Us7*, and *Us8*. The green box represents the CMV promoter. The red box represents the mCherry ORF. Diagrams are not to scale.

For our pathogenesis studies of HSV-1, we produced and characterized a recombinant strain of HSV-1 that behaves like the wild-type parental strain KOS, and that stably expresses the second generation red fluorescent protein (RFP) mCherry [39] (Figure 1). This RFP was chosen due to its stability and the fact that it functions as a true monomer. Furthermore, due to the inherent autofluorescence of neural tissue in the green spectrum, the use of an RFP minimized background signal from uninfected cells in the TG. The cloning strategy chosen was designed so as not to disrupt any viral open reading frames (ORFs) or regulatory regions of the surrounding genes. An mCherry expression cassette under control of a mammalian promoter was inserted into the intergenic region between the ORFs of the HSV-1 *Us7* and *Us8* genes. To avoid disrupting the polyadenylation of transcripts co-terminal with *Us7*, as well as to avoid interrupting the promoter of *Us8*, the *Us7* and *Us8* intergenic region was duplicated in tandem. Two independent isolates of the recombinant virus vUs7-8mCherry, a and b, were produced by homologous recombination.

### Characterization of vUs7-8mCherry *in vitro* and *in vivo*

**Figure 2:**
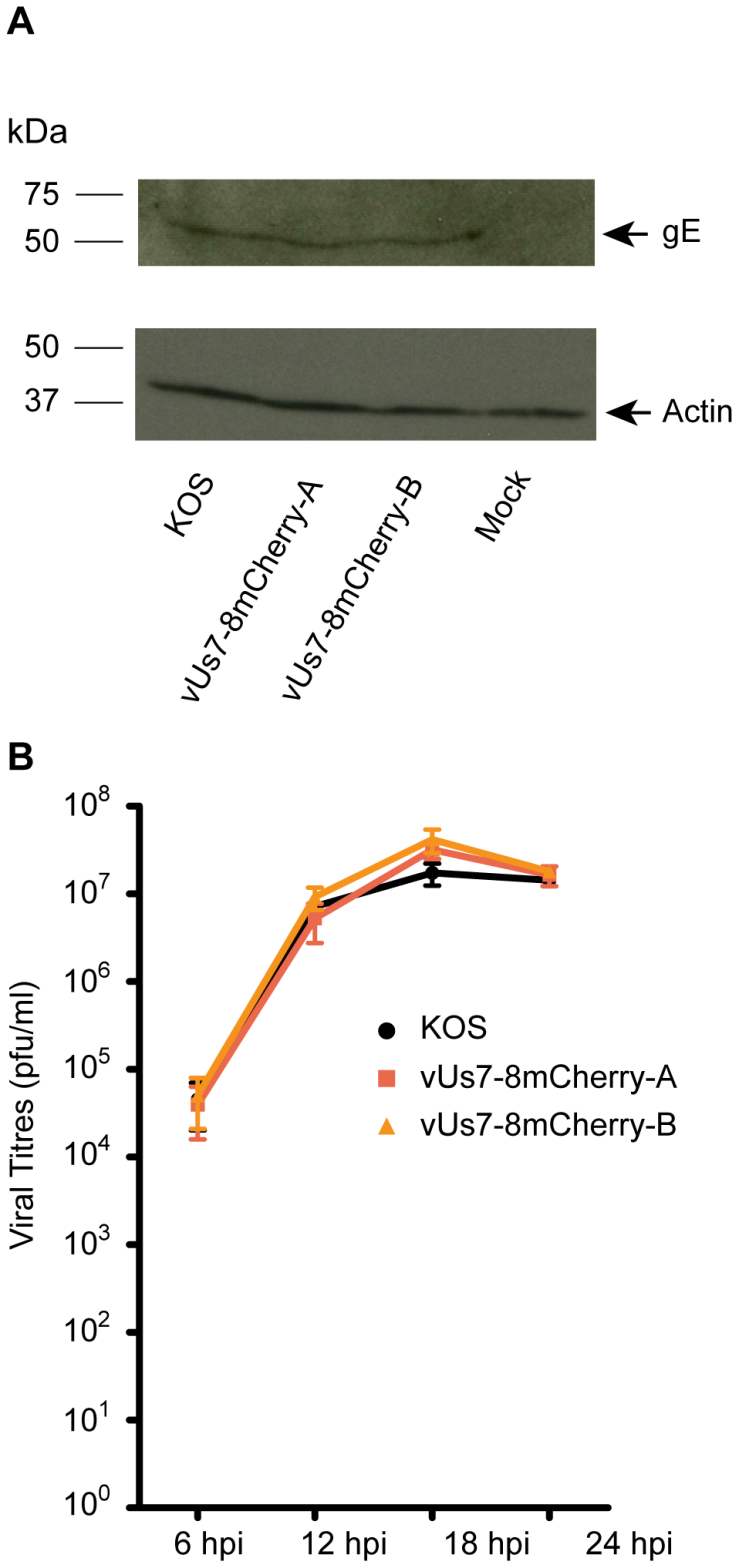
Characterization of vUs7-8mCherry. (A) Western blot showing expression of the viral protein Us8 (gE) in Vero cells infected by vUs7-8mCherry (isolates a and b) (top panel). The blot was stripped and reprobed for beta-Actin as a loading control (bottom panel). The positions of molecular mass markers are indicated to the left of each panel. Arrows to the right of the panels mark the position of the protein. (B) One-step replication analysis of vUs7-8mCherry (a and b) compared to the wild-type virus KOS. Anova statistical tests revealed no significant differences in the values obtained for vUs7-8mCherry and KOS.

Prior to using vUs7-8mCherry for our experiments, we tested whether the virus behaved like the parental strain both in cell culture and *in vivo*. For several HSV-1 strains in which genes for intrinsically fluorescent proteins have been inserted into the genome, stability has proven to be a problem and expression cassettes were either repressed or lost due to the recombinegetic nature of the virus [4], [40]. In contrast, we found that vUs7-8mCherry was very stable; the mCherry expression cassette was maintained by the virus throughout multiple rounds of replication *in vitro* (data not shown). We also tested the impact of the insertion of the mCherry cassette on expression of the downstream gene *Us8*. *Us8* codes for the viral glycoprotein E (gE). To confirm that our strategy was successful in preserving wild type expression levels of gE, we assessed steady-state levels of the glycoprotein in Vero cells infected for 18 hours with vUs7-8mCherry (Figure 2 A). By Western blot analysis, we found that gE protein levels were similar for each of the isolates and the parental strain KOS. Furthermore, we found that both isolates replicated as efficiently as the wild-type virus KOS in a single-step replication assay (Figure 2 B).

**Figure 3:**
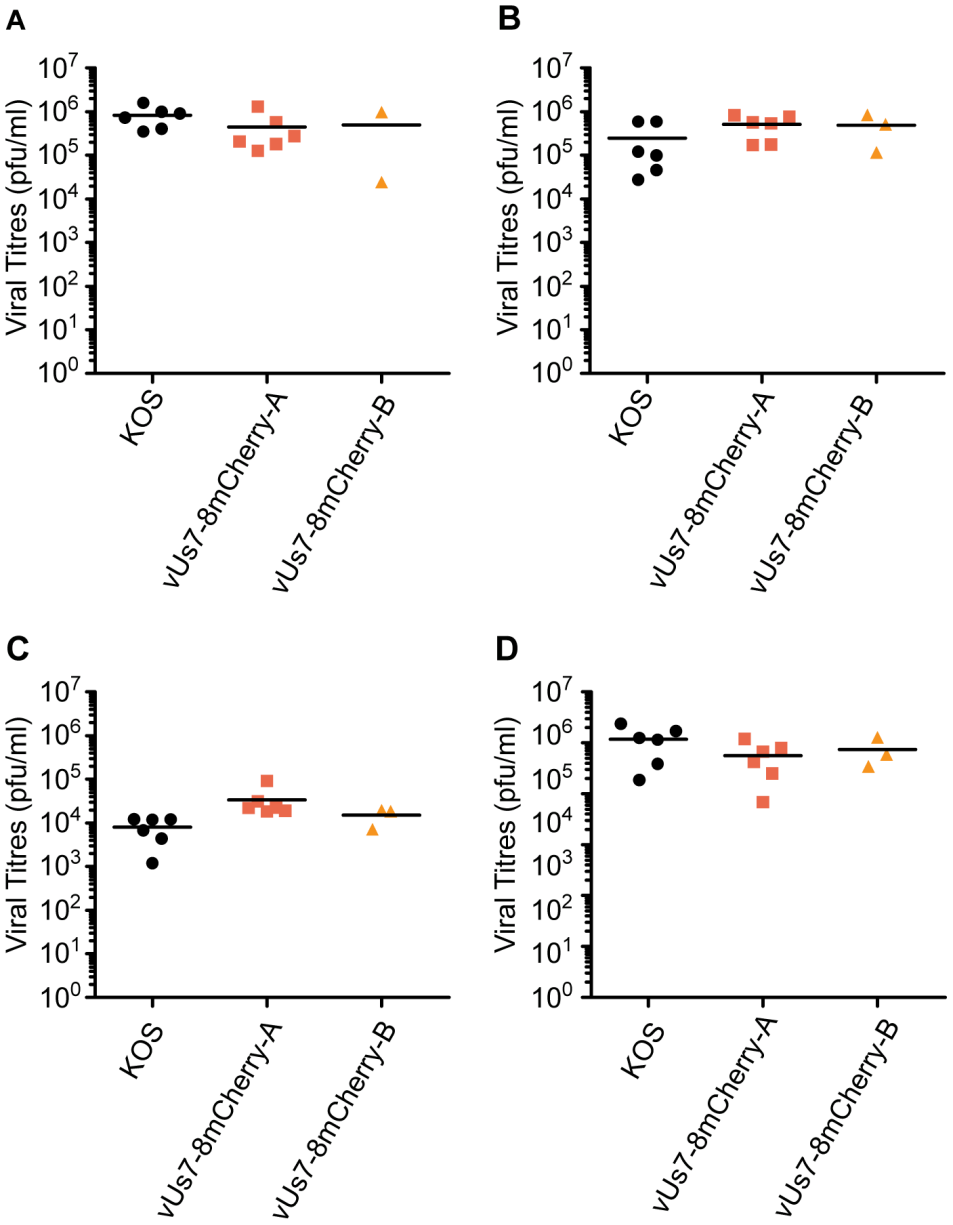
Insertion of the mCherry expression cassette between *Us7* and *Us8* does not alter virus phenotype *in vivo*. Both independent isolates of vUs7-8mCherry were tested *in vivo* in parallel with the wild-type virus KOS as a control. Titers of virus present in tear films of mice at 1 (A), 2 (B) and 3 (C) days p.i. (D) Titers of virus present in the TG of mice harvested at 3 days p.i. Anova statistical tests revealed no significant differences in the values obtained for vUs7-8mCherry and KOS.

We next assessed the efficiency of vUs7-8mCherry to replicate *in vivo* using a well-established murine model of ocular infection [32]. Mice were infected with 2×10^6^ plaque forming units (pfu) of HSV-1 per eye following light scarification of the cornea. For the first three days post infection (dpi), both isolates of vUs7-8mCherry produced viral titres in tear films similar to KOS (Figure 3 A-C). Likewise, at three dpi, the viral titres in TG for vUs7-8mCherry were similar to wild-type levels (Figure 3 D). We next tested the ability of vUs7-8mCherry to reactivate from latently infected TG in an explant assay. In this assay, TG are dissociated enzymatically, and then co-cultured on a monolayer of Vero cells. By 24 hours post dissociation, mCherry-positive neurons were readily detected using fluorescence microscopy (data not shown). Furthermore, no reduction in the frequency of reactivation as compared to KOS was observed for either independent isolate of vUs7-8mCherry (Table 1). In addition, we found that vUs7-8mCherry was stable *in vivo*, because we did not observe loss of mCherry expression following passage through mice either during acute replication or following *ex vivo* reactivation (data not shown).

**Table 1:** Expression of mCherry does not reduce viral *ex vivo* reactivation frequency.

Virus	# TG reactivated	(%)	
	TG dissociated		
KOS	10/14	(86%)	
Mock	0/4	(0%)	*
vUs7-8mCherry-a	10/10	(100%)	†
vUs7-8mCherry-b	8/8	(100%)	†

*: p< 0.0001 compared to KOS (Fisher's exact test).

† : p>0.1 compared to KOS (Fisher's exact test).

### Visualisation of infected cells by fluorescence microscopy

**Figure 4:**
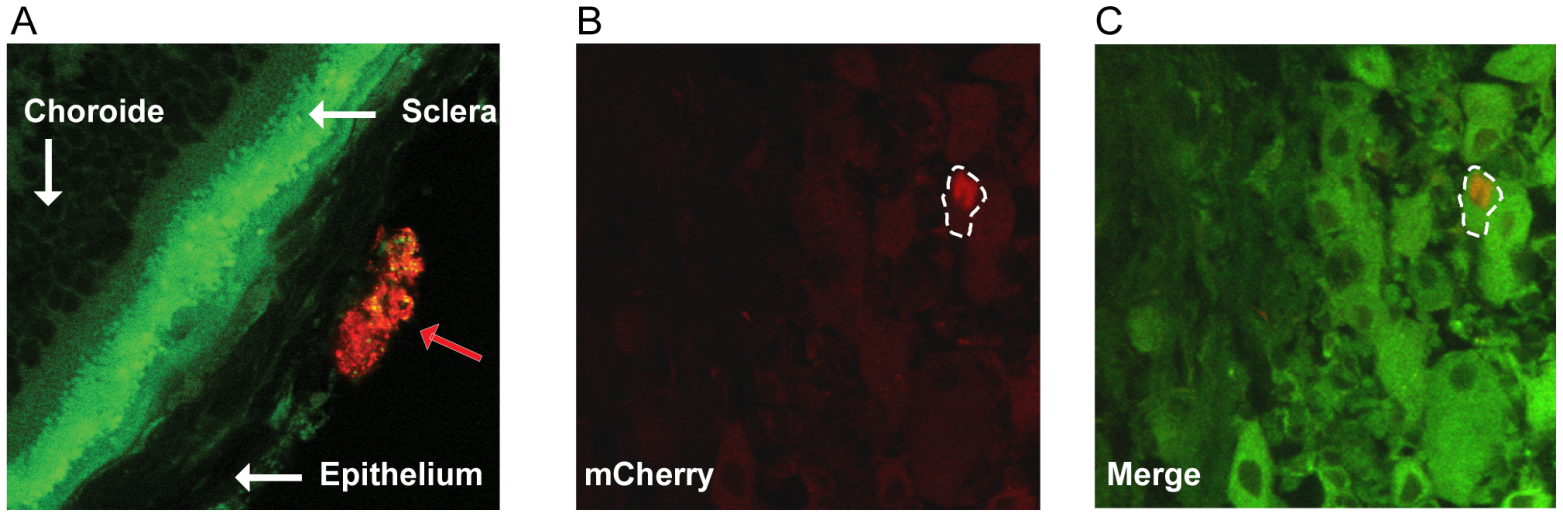
Identification of cells infected by vUs7-8mCherry in histological sections. (A) Eyes of mice infected with vUs7-8mCherry-a were harvested at 2 days p.i., and analyzed by standard confocal microscopy. Structures of the eye are indicated by the white arrows. The red arrow indicates a group of cells infected by vUs7-8mCherry-a. (B) TG of mice infected with vUs7-8mCherry were harvested at 3 days p.i.; an infected neuron is visualized by confocal microscopy using the red field. (C) Merge image showing the autofluorescence of the TG in green field and the red field showing an infected cell. The white dashed line outlines an infected neuron.

Prior to imaging of infected tissues using our multimodal multiphoton platform, we assessed our ability to visualize vUs7-8mCherry-infected tissues by standard confocal microscopy. Eyeballs and TG were harvested post-mortem at two and three dpi. Tissues were fixed and processed for sectioning by cryostat, and 10 µm sections were mounted on microscope slides (Figure 4). Many subanatomical structures were readily identifiable by their autofluorescence. The opaque sclera of the eye, as well as the clusters of pseudomonopolar neurons in the TG, naturally generate a very bright signal in the green field due to their richness in epithelial cell NADPH and cellular matrix collagen, and in lipofuscin respectively [19], [21], [41]. In contrast, autofluorescence of these tissues drops dramatically once in the red field such that corneal epithelial cells infected by vUs7-8mCherry stood out from the background (Figure 4 A). Similarly, in the TG, infected neurons were identifiable via mCherry fluorescence, which was also above the autofluorescence background signal in the red field (Figure 4 B-C). We next confirmed that we could identify the same infected region using fluorescence microscopy to visualize mCherry expressed by our recombinant virus, as we could by standard immunohistochemistry. Samples were processed for immunohistofluorescence using antiserum directed against HSV-1 and a secondary antibody conjugated to Alexa 488 (Figure S1). As expected, the subcellular structures visualized by each approach differed. The soluble protein mCherry was detected in the cytoplasm and nucleus. In contrast, the HSV-1 antiserum, which contained many antibodies directed against viral glycoproteins, stained mainly cell membranes and cytoplasmic structures. Nevertheless, the same region of the TG that was stained using the HSV-1 antiserum was detected by imaging mCherry expression, thus validating our strategy to use a virus that expresses a red fluorescent protein for detection with our platform.

### Visualization of whole TG using multimodal non-linear optical microscopy

**Figure 5:**
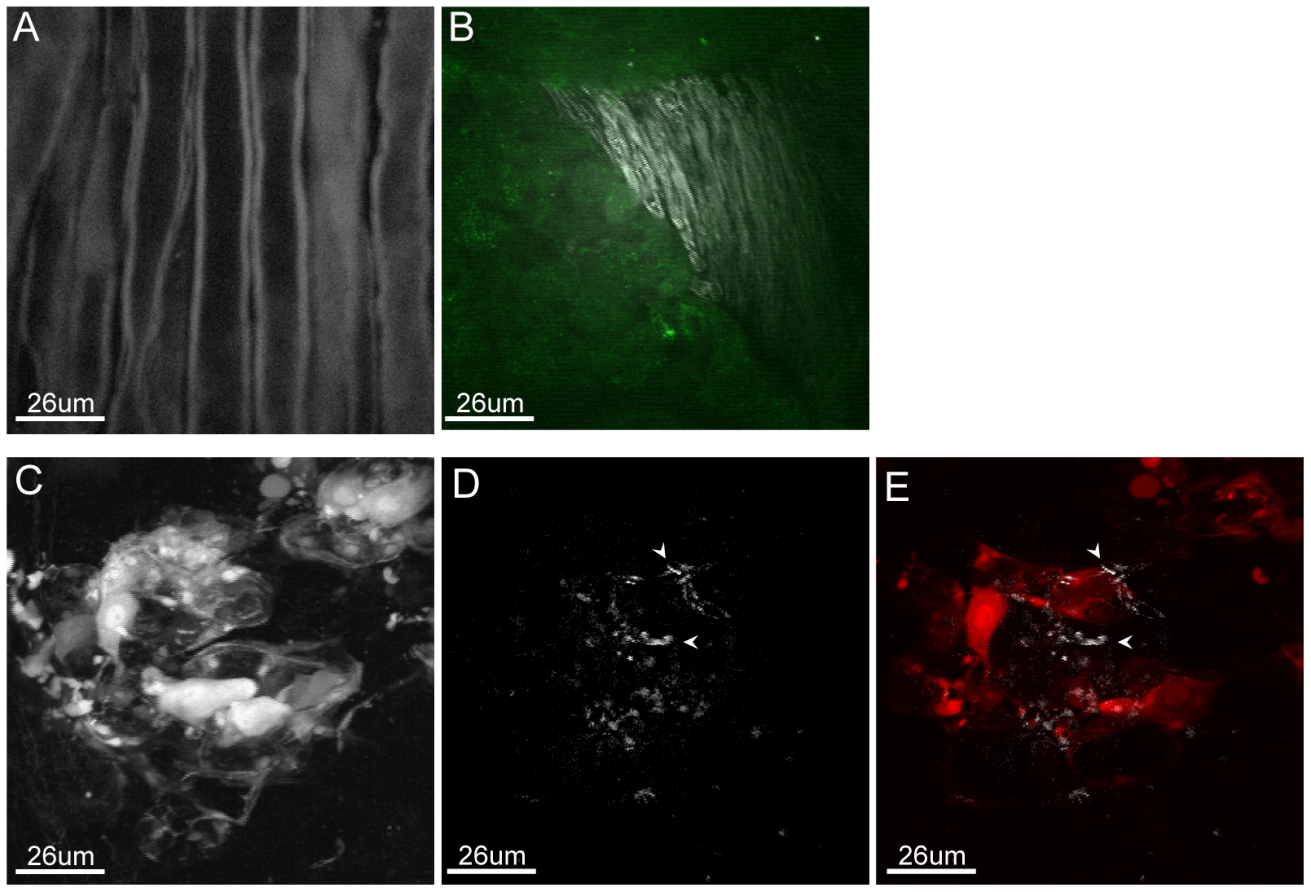
Visualization of a non-sectioned infected TG with a multimodal non-linear imaging platform. (A) Myelinated axons as visualized by CARS microscopy. (B) Merge of the CARS signal (white) with the autofluorescence of tissue corresponding to a cluster of neuron bodies (green). (C) Visualization of infected cells through detection of mCherry fluorescence. The image represents the sum of all focal planes taken at 2 microns interval through the TG. (D) CARS imaging of one of the focal planes visualized in (C) from which the autofluorescence background was subtracted. (E) Merge of mCherry fluorescence (red) with the corresponding focal plane of CARS microscopy (white) in (D). White arrowheads indicate a myelinated axon visualized by CARS microscopy.

One of the major advantages of two-photon fluorescent microscopy compared to traditional confocal microscopy is the ability to conduct deep tissue imaging. We next visualized sites of viral replication in whole, unfixed, infected TG using our multimodal non-linear optical microscopy platform (Figure 5). Mock-infected TGs as well as vUs7-8mCherry-infected TGs were harvested at 3 dpi, and immediately placed in ice cold PBS. The delay between time of harvest and analysis was maintained to less than 24 hours to preserve tissue architecture. TGs used for these analyses were neither fixed nor sectioned. Following harvest, a typical TG is approximately 1 mm in diameter and 1 cm in length. For imaging purposes, TG were placed on a microscope slide, overlayed with a small amount of cold PBS, and covered with a no. 1 coverslip.

Phospholipids that make up the cellular plasma membrane are rich in symmetric CH_2_ vibrational modes. Thus, the myelin sheath surrounding neuronal axons, which are formed by multiple layers of Schwann cell plasma membrane, are readily visualized by CARS microscopy. Although we noted that some autofluorescence signal could be detected when using the filter for CARS microscopy, we were able to clearly visualize axonal extensions and distinguish between the inside and the borders of the axons (Figure 5 A). By combining CARS microscopy with 2-photon fluorescence microscopy, which allowed us to visualize neuronal cell bodies by autofluorescence in the green spectrum, we were able to distinguish between regions dominated by axonal extensions and regions containing clusters of non-infected pseudomonopolar neurons (Figure 5 B).

We next attempted to image infected cells in their three dimensional tissular context using our microscopy platform. For imaging of intact TG, infected sites within TG were first located by two-photon fluorescent microscopy using a filter for mCherry. Cells infected with vUs7-8mCherry were readily identified deep within the TG. Through the collection of a series of Z-stacks, we reconstructed a three-dimensional projection of a cluster of infected neurons visualized through the detection of mCherry using the program NIH Image J (Figure 5 C and Video S1). Once an infected region of the TG was identified, we selected one of the focal planes to visualize using both CARS microscopy alone or combined with fluorescence microscopy (Figure 5 D, E respectively). The autofluorescence signal was digitally substracted from our CARS field images to put emphasis on the signal generated by myelinated axons (Figure 5 D). This particular region, dominated by neuronal cell bodies, generated CARS signal due to axons located between the cell bodies. The focal plane visualized by CARS microscopy was merged with the corresponding field obtained from two-photon microscopy (Figure 5 E). Thus, we successfully combined two-photon fluorescence microscopy with CARS imaging to visualize HSV-1-infected neurons deep in unfixed and unsectioned neurons within TG.

## Discussion

Although there has been much written about the potential of tissue imaging with multimodal multiphoton microscopy [20], [42], to the best of our knowledge, this study constitutes the first report of combining CARS microscopy and two-photon fluorescence microscopy to study viral infections deep within whole tissue mounts. Combining the two techniques enabled us to visualize distinct subanatomical structures within the TG, both in infected and uninfected regions of interest (ROI). Regions of dense bundles of myelin rich axons within TG were readily observed by CARS microscopy. In contrast, regions corresponding to large clusters of neuronal cell bodies produced smaller areas of CARS signal in between the cell bodies. When using the fluorescence imaging modality, the multimodal non-linear optical microscopy platform enabled us to visualize uninfected bodies deep within TG by virtue of their autofluorescence in the green spectrum, as well as infected cells via the expression of mCherry by the virus.

Although there have been numerous HSV-1 strains constructed that express intrinsically fluorescent proteins, few have been generated that avoid disrupting any viral ORF, and that maintain a wild-type phenotype both *in vitro* and *in vivo* [4], [43], [44], [45], [46], [47], [48]. Furthermore, some were demonstrated to be unstable leading to loss of expression of the fluorescent protein after several passages [4]. We have successfully engineered a recombinant HSV-1 strain that expresses mCherry all the while retaining a wild-type phenotype *in vivo*. Furthermore, we found that the virus is stable both in cell culture and following passage through mice. Thus, this virus constitutes one of a limited number of recombinant HSV-1 strains that encode an intrinsically fluorescent protein, and that are suitable for *in vivo* studies looking at pathogenesis [49], [50].

mCherry was an excellent choice for visualization of infected cells *in situ* due to the low natural fluorescence of biological samples corresponding to its visual spectrum. The mCherry signal was sufficiently above the autofluorescence background with limited photobleaching to detect both the cell bodies and the axons of many infected neurons in the tissue. Further improvement to our strategy would include having an even stronger signal to noise ratio for the multiphoton fluorescent imaging. One approach would be the use of the RFP tdTomato, for which the gene is a duplicated mTomato ORF in tandem and in frame. This intramolecular dimer is three times more brilliant than mCherry, and has been detected as deep as 1 cm within tissues, making it ideal for the study of viral dissemination within an experimental animal [51].

Finally, using the sectioning power of our microscopy platform, we recreated a three-dimensional projection of an ROI deep within the unfixed TG where infected neurons were located. In this projection, infected neurons and axons could be observed in relation to their native environment. From there, we were able to select ROIs to analyze in more detail combining fluorescence and CARS microscopy techniques. Our results demonstrate the potential of multimodal non-linear optical microscopy for studying viral infection and pathogenesis in whole tissues by combining data acquired through different imaging modalities. Furthermore, because CARS imaging is particularly well suited to studying demyelinating diseases, our platform would be ideal for investigating hypotheses of virus-induced demyelination among other virus-induced neuropathologies. Although our experiments were conducted on explanted tissue, our ability to accomplish this imaging without fixatives, stains or sectioning of the tissue demonstrates the potential for applications in *in vivo* imaging. Future improvements to the system include strategies to increase the signal to background ratio of CARS signals in biological samples, and the use of brighter intrinsically fluorescent proteins that, nevertheless, do not emit at a wavelength corresponding to high autofluorescence in different tissues.

## Materials and Methods

### Ethics Statement

All animal experiments were carried out at the INRS Centre for Biological Experimentation in accordance with institutional good animal care practices, and approved by the "Comité institutionnel de protection des animaux" of Université INRS (permit #0806-04).

### Plasmid construction

To create a eukaryotic expression cassette for mCherry, the ORF was excised from the prokaryotic expression vector pRSETb-mCherry [39] with HindIII and BamHI. The resulting fragment was inserted within the multiple cloning site of a pCG-Zeo derived eukaryotic expression vector [52] containing the CMV promoter, producing the plasmid pCG-mCherry.

To minimize the impact on expression of the neighbouring genes, the *Us7* and *Us8* intergenic region was duplicated in tandem separated by a unique PmeI site. Each fragment was produced by PCR using KOS DNA as a template. The 5′ fragment was obtained using the primers 5′-cccggatccctaatgccacgcgagcg and 5′-ggggtttaaacgagccaaagtcaacacaac. The 3′ fragment was obtained using the primers 5′-cccgtttaaactgtccatttctttcttccc and 5′-cccaagcttgcgagactttcgtcctc. The 5′ and 3′ PCR fragments were digested with PmeI and either BamHI or HindII respectively. By triple ligation using T4 DNA ligase (NEB), the fragments were inserted within the pET15b plasmid that had been digested with BamHI and HindIII. The resulting plasmid, pET15b/*Us7-Us8,* was verified by standard DNA sequencing. Sequencing was carried out by the McGill University Genome Quebec Innovation Centre.

To produce the transfer vector, we next amplified the mCherry expression cassette from the plasmid pCG-mCherry using the primers 5′-ccgcggatagaattcgag and 5′-ccgcggatacgaattcttac. The plasmid pET15b/*Us7-Us8* was digested with the restriction enzyme PmeI, creating a site in which to insert the expression cassette by blunt ligation. The resulting plasmid was verified by DNA sequencing.

### Construction of recombinant viruses

Recombinant viruses were produced by homologous recombination essentially as described previously [53]. The virus vUs7-8mCherry was obtained by co-transfecting KOS infectious DNA and the transfer vector pET15b/*Us7-Us8*mCherry that had been linearized with HindIII. DNA was transfected into Vero cells using Lipofectamine (Life Technologies) according to the manufacturer's instructions. Screening of recombinant viruses was based on expression of mCherry. Two independent isolates, originating from two independent transfections, were produced (a and b). The *Us7* and *Us8* intergenic regions of each independent isolate were sequenced to ensure the absence of undesired mutations.

### Viruses and cells

KOS and vUs7-8mCherry isolates were propagated on Vero cells as described previously [54]. The KOS strain was originally from the Priscilla Schaffer lab (Harvard Medical School). Cells were grown in DMEM containing 5% newborn calf serum (NCS), and the antibiotics penicillin and streptomycin, and maintained in a 37°C incubator with 5% CO_2_.

### Western Blotting

Vero cells were infected with either KOS or either isolate of vUs7-8mCherry at a multiplicity of infection (MOI) of 5. At 18 hours post-infection (hpi), cells were lysed in RIPA buffer (50 mM Tris pH 7.5, 1% Triton X-100, 0.5% DOC, 0.1% SDS, 500 mM NaCl). Proteins were resolved by SDS polyacrylamide gel electrophoresis, and transferred to a polyvinylidene difluoride membrane (Immobilon-P; Millipore). Proteins were detected by Western blotting using a mouse monoclonal antibody directed against HSV-1 gE (Fitzgerald) or a mouse monoclonal antibody directed against beta-Actin (Biolegend), followed by appropriate secondary antibodies conjugated to horseradish peroxidise (Bethyl and Sigma respectively). Detection was done by enhanced chemiluminescence (GE) according to the manufacturer's instructions.

### Viral replication assays

Viral yield was determined using one-step replication assays. Vero cells (2.5×10^5^) were seeded in duplicate in cell culture tubes containing 2 ml of complete medium. The next day, cells were infected with the indicated virus at an MOI of 5. At 6, 12, 18, and 24 hpi, the respective tubes were removed from the incubator and placed at −80°C. Tubes were thawed and sonicated, and total virus (cell associated and cell free) was titrated.

### Murine model of ocular infection

Mouse experiments were conducted essentially as described previously [32], [55]. Seven-week old CD-1 mice were obtained from Charles River, and were acclimatized for 5–7 days. Prior to infection, mice were deeply anaesthetized by intraperitoneal injection of a solution of ketamine (100 mg kg^−1^; Bioniche) and xylazine (10 mg kg^−1^; Bayer) diluted in saline. Following light scarification of the cornea, 2×10^6^ plaque forming units (pfu) of virus were applied in a dropwise manner in a volume of 7–10 µL to each cornea of the mice. Groups of at least five animals per virus were infected in each individual experiment. Virus titres during acute eye infection were determined from 1 to 3 d.p.i. by titrating virus in pooled tear films from both eyes collected with moistened sterile cotton swabs while the mice were anaesthetized with isofluorane (Baxter). Titres of virus in TG during acute infection were determined by titrating virus present in the pooled homogenates of both TG harvested post-mortem at 3 d.p.i. For titres in the eye, on each day in question, eye swabs were taken from three mice per virus. For titres in TG, TG were harvested from three mice for each virus per experiment. Virus reactivation following latency was assessed in an explant assay 30–40 days p.i. Individual TG were dissociated with collagenase (Invitrogen) and overlaid on a monolayer of Vero cells. Reactivation for each TG was determined by the appearance of the virus-induced cytopathic effect of the Vero cells, which was monitored for 10 days post-explant.

### Tissue sections and confocal analysis

Eyeballs harvested 2 d.p.i., and TG harvested at 3 d.p.i., were fixed for 24 h in 4% PFA at 4°C, and stored in PBS. Prior to sectioning, samples were placed in 30% sucrose in PBS overnight at 4°C, immersed in frozen tissue embedding media Histo Prep (fisher Scientific), and frozen on dry ice until the mould was solidified. Serial 10 µM sections were prepared using a cryostat (Kryostat 1720 digital; Leitz, Midland), and mounted on Superfrost Plus slides (Fisher Scientific). Cover slips were mounted using Prolong Gold antifade reagent (Invitrogen). The samples were visualized using a Bio-Rad Radiance 2000 confocal system with an argon-krypton laser at 488 and 568 nm (diode, 638 nm) mounted on a Nikon E800 microscope. Images were prepared using Adobe Photoshop CS4 software.

### Immunohistofluorescence analysis of TG sections

Detection of infected cells in histological sections was achieved by immunohistofluorescence (IHF). Immediately following tissue sectioning, samples were permeabilized for 30 minutes with PBS containing 5% NCS and 0.1% Triton X-100. Slides were dried briefly using absorbent paper, and incubated overnight at 4°C in a humid chamber (Sigma) with a rabbit polyclonal anti-HSV-1 (AbCam) primary antibody at a dilution of 1∶150, and Hoechst (Invitrogen life technologies) at a dilution of 1∶500 for nuclear staining. The next day, slides were washed three times with PBS containing 5% NCS, and incubated for 1.5 hours at room temperature with a goat polyclonal anti-rabbit IgG antibody conjugated to Alexa488 diluted 1∶1000. All antibodies were diluted in PBS containing 5% NCS. Three additional washes were then carried out before mounting slide covers using ProlongGold antifade reagent (Invitrogen life technologies). The samples were visualized using a Zeiss Axio Observer Z1 (63X, N.A.1.4.) microscope with a diode laser at 405 nm, an argon multi-line laser at 458/488/514 nm, a DPSS laser at 561 nm, and a HeNe laser at 633 nm. Images were prepared using Adobe Photoshop CS4 software.

### Multimodal non-linear optical microscopy platform

For the multiphoton microscopy, we used a laser scanning inverted microscope (Till Photonics GmbH, Munich, Germany). The focusing objective used in our experiments was the Olympus UAPO 40XW3/340 (water immersion with a numerical aperture of 1.15). The size of the laser beams at the entrance of the XY scanner was set to ensure that the aperture of the focusing objective was completely illuminated. Its position relative to the sample was controlled by a mechanic and a piezoelectric motor for coarse and fine adjustment. Because the imaged samples were thick, the detection was performed in the backward direction. CARS and two-photon fluorescence light was then collected with the same microscope objective, and redirected to a PMT detector (model R6357, Hamamatsu Corporation, New Jersey, USA) by a dichroic mirror (model FF735-Di01-25, Semrock, Rochester, USA). Additional filters are put in front of the detector to eliminate residual laser beams and isolate the desired signal.

For CARS microscopy, a ∼150 fs pulse duration Titanium-Sapphire oscillator (Tsunami, Spectra Physics, Santa Clara, USA) was spatially and temporally overlapped with a 15 ps pulse duration Nd:YVO_4_ laser (Vanguard, Spectra Physics, Santa Clara, USA). The two lasers were electronically locked to have the same repetition rate (∼80 MHz). The wavelength of the Titanium-Sapphire (817 nm) and of the Nd:YVO_4_ (1064 nm) laser were set in order that their frequency difference met the symmetric CH_2_ vibrational mode (∼2845 cm-1) [17], [42]. Before being overlap with the Nd:YVO_4_ laser, the bandwidth of the Titanium-Sapphire spectra was reduced through a pulse shaper to around 1 nm to obtain a pulse duration of around 1 ps thus increasing the spectral resolution and the ratio of the resonant CARS signal to the non-resonant CARS background [56], [57], [58].

For two-photon fluorescence microscopy [59], [60], a ∼130 fs pulse duration optical parametric oscillator (OPO) pumped by the Titanium-Sapphire oscillator was used (Opal, Spectra Physics, Santa Clara, USA). We set the output wavelength at 1180 nm to be as close as possible to the 2-photon excitation peak of the mCherry fluorophore [39]. Due to the fact that the Titanium-Sapphire wavelength used for the OPO pumping (775 nm) is not the same as the one for CARS microscopy, two-photon excitation and CARS microscopy techniques were used alternately.

## Supporting Information

Figure S1 **Identification of cells infected with vUs7-8mCherry in TG sections by visualization of mCherry fluorescence and by anti-HSV-1 immunohistofluorescence.** TG from mice infected with vUs7-8mCherry were harvested 3 dpi, sectioned, immunostained for HSV-1, and analyzed by confocal microscopy. Shown are infected cells as visualized by immunohistofluorescence using anti-HSV-1 serum (top left panel), and by detection of mCherry fluorescence (top right panel). Nuclei were stained with Hoechst, which labels DNA (bottom left panel). Merged images are shown in the bottom right panel. Arrows point to nuclei of infected cells, arrowheads point to nuclei of non-infected cells. The scale bar represents 20 µm.
(TIFF)

Video S1 **Three dimensional reconstitution through two-photon fluorescence microscopy of neurons within TG infected with vUs7-8mCherry.** Cells within a trigeminal ganglion infected with vUs7-8mCherry were visualized by two-photon fluorescence microscopy. Images were taken at every 2 microns using optical sectioning, for a total depth of 88 microns. Using NIH image J software with signal interpolation, the 44 images taken were used to reconstruct a three-dimensional image of a region of interest.
(AVI)

## References

[1] Sillevis Smitt PA, van der Loos C, Vianney de Jong JM, Troost D (1993) Tissue fixation methods alter the immunohistochemical demonstrability of neurofilament proteins, synaptophysin, and glial fibrillary acidic protein in human cerebellum. Acta Histochem 95: 13–21. 7506474 10.1016/s0065-1281(11)80381-8

[2] Luker GD, Bardill JP, Prior JL, Pica CM, Piwnica-Worms D, et al (2002) Noninvasive bioluminescence imaging of herpes simplex virus type 1 infection and therapy in living mice. J Virol 76: 12149–12161. 12414955 10.1128/JVI.76.23.12149-12161.2002 PMC136903

[3] Luker GD, Prior JL, Song J, Pica CM, Leib DA (2003) Bioluminescence imaging reveals systemic dissemination of herpes simplex virus type 1 in the absence of interferon receptors. J Virol 77: 11082–11093. 14512556 10.1128/JVI.77.20.11082-11093.2003 PMC224994

[4] Balliet JW, Kushnir AS, Schaffer PA (2007) Construction and characterization of a herpes simplex virus type I recombinant expressing green fluorescent protein: acute phase replication and reactivation in mice. Virology 361: 372–383. 17207829 10.1016/j.virol.2006.11.022 PMC1975764

[5] Brown CP, Houle MA, Chen M, Price AJ, Legare F, et al (2012) Damage initiation and progression in the cartilage surface probed by nonlinear optical microscopy. J Mech Behav Biomed Mater 5: 62–70. 22100080 10.1016/j.jmbbm.2011.08.004

[6] Gusachenko I, Tran V, Goulam Houssen Y, Allain JM, Schanne-Klein MC (2012) Polarization-resolved second-harmonic generation in tendon upon mechanical stretching. Biophys J 102: 2220–2229. 22824287 10.1016/j.bpj.2012.03.068 PMC3341536

[7] Schurmann S, von Wegner F, Fink RH, Friedrich O, Vogel M (2010) Second harmonic generation microscopy probes different states of motor protein interaction in myofibrils. Biophys J 99: 1842–1851. 20858429 10.1016/j.bpj.2010.07.005 PMC2941014

[8] Stoothoff WH, Bacskai BJ, Hyman BT (2008) Monitoring tau-tubulin interactions utilizing second harmonic generation in living neurons. J Biomed Opt 13: 064039. 19123685 10.1117/1.3050422 PMC3004129

[9] Andreas V (2005) Vibrational imaging and microspectroscopies based on coherent anti-Stokes Raman scattering microscopy. Journal of Physics D: Applied Physics 38: R59.

[10] Mostaço-Guidolin LB, Sowa MG, Ridsdale A, Pegoraro AF, Smith MSD, et al (2010) Differentiating atherosclerotic plaque burden in arterial tissues using femtosecond CARS-based multimodal nonlinear optical imaging. Biomedical Optics Express 1: 59–73. 21258446 10.1364/BOE.1.000059 PMC3005156

[11] Volkmer A, Cheng J-X, Sunney Xie X (2001) Vibrational Imaging with High Sensitivity via Epidetected Coherent Anti-Stokes Raman Scattering Microscopy. Physical Review Letters 87: 023901.

[12] Evans CL, Potma EO, Puoris'haag M, Cote D, Lin CP, et al (2005) Chemical imaging of tissue in vivo with video-rate coherent anti-Stokes Raman scattering microscopy. Proc Natl Acad Sci U S A 102: 16807–16812. 16263923 10.1073/pnas.0508282102 PMC1283840

[13] Folick A, Min W, Wang MC (2011) Label-free imaging of lipid dynamics using Coherent Anti-stokes Raman Scattering (CARS) and Stimulated Raman Scattering (SRS) microscopy. Curr Opin Genet Dev 21: 585–590. 21945002 10.1016/j.gde.2011.09.003 PMC3206170

[14] Fu Y, Wang H, Huff TB, Shi R, Cheng JX (2007) Coherent anti-Stokes Raman scattering imaging of myelin degradation reveals a calcium-dependent pathway in lyso-PtdCho-induced demyelination. J Neurosci Res 85: 2870–2881. 17551984 10.1002/jnr.21403 PMC2277477

[15] Hellerer T, Axang C, Brackmann C, Hillertz P, Pilon M, et al (2007) Monitoring of lipid storage in Caenorhabditis elegans using coherent anti-Stokes Raman scattering (CARS) microscopy. Proc Natl Acad Sci U S A 104: 14658–14663. 17804796 10.1073/pnas.0703594104 PMC1976189

[16] Lim RS, Suhalim JL, Miyazaki-Anzai S, Miyazaki M, Levi M, et al (2011) Identification of cholesterol crystals in plaques of atherosclerotic mice using hyperspectral CARS imaging. J Lipid Res 52: 2177–2186. 21949051 10.1194/jlr.M018077 PMC3220286

[17] Wang H, Fu Y, Zickmund P, Shi R, Cheng JX (2005) Coherent anti-stokes Raman scattering imaging of axonal myelin in live spinal tissues. Biophys J 89: 581–591. 15834003 10.1529/biophysj.105.061911 PMC1366558

[18] Lyn RK, Kennedy DC, Sagan SM, Blais DR, Rouleau Y, et al (2009) Direct imaging of the disruption of hepatitis C virus replication complexes by inhibitors of lipid metabolism. Virology 394: 130–142. 19747705 10.1016/j.virol.2009.08.022

[19] Teng SW, Tan HY, Peng JL, Lin HH, Kim KH, et al (2006) Multiphoton autofluorescence and second-harmonic generation imaging of the ex vivo porcine eye. Invest Ophthalmol Vis Sci 47: 1216–1224. 16505061 10.1167/iovs.04-1520

[20] Lin CY, Hovhannisyan V, Wu JT, Lin CW, Chen JH, et al (2008) Label-free imaging of Drosophila larva by multiphoton autofluorescence and second harmonic generation microscopy. J Biomed Opt 13: 050502. 19021374 10.1117/1.2981817

[21] Mochizuki Y, Park MK, Mori T, Kawashima S (1995) The difference in autofluorescence features of lipofuscin between brain and adrenal. Zoolog Sci 12: 283–288. 7580812 10.2108/zsj.12.283

[22] Roizman B, Knipe DM, Whitley RJ (2007) Herpes Simplex Viruses. In: Knipe DM, editor. Fields Virology: Lippincott Williams & Wilkins. pp. 3177.

[23] Roizman B, Furlong D (1974) The replication of herpes viruses. Comprehensive Virology 3: 175.

[24] Blyth WA, Harbour DA, Hill TJ (1984) Pathogenesis of zosteriform spread of herpes simplex virus in the mouse. J Gen Virol 65 (Pt 9): 1477–1486. 6088680 10.1099/0022-1317-65-9-1477

[25] Summers BC, Margolis TP, Leib DA (2001) Herpes simplex virus type 1 corneal infection results in periocular disease by zosteriform spread. J Virol 75: 5069–5075. 11333887 10.1128/JVI.75.11.5069-5075.2001 PMC114911

[26] Cook ML, Stevens JG (1973) Pathogenesis of herpetic neuritis and ganglionitis in mice: evidence for intra-axonal transport of infection. Infect Immun 7: 272–288. 4348966 10.1128/iai.7.2.272-288.1973 PMC422671

[27] Baringer JR, Swoveland P (1973) Recovery of herpes-simplex virus from human trigeminal ganglions. N Engl J Med 288: 648–650. 4347057 10.1056/NEJM197303292881303

[28] Reichelt M, Zerboni L, Arvin AM (2008) Mechanisms of varicella-zoster virus neuropathogenesis in human dorsal root ganglia. J Virol 82: 3971–3983. 18256143 10.1128/JVI.02592-07 PMC2292995

[29] Dyson H, Shimeld C, Hill TJ, Blyth WA, Easty DL (1987) Spread of herpes simplex virus within ocular nerves of the mouse: demonstration of viral antigen in whole mounts of eye tissue. J Gen Virol 68 (Pt 12): 2989–2995. 2826644 10.1099/0022-1317-68-12-2989

[30] Kimberlin D (2004) Herpes simplex virus, meningitis and encephalitis in neonates. Herpes 11 Suppl 265A–76A. 15319092

[31] Pottage JC Jr, Kessler HA (1995) Herpes simplex virus resistance to acyclovir: clinical relevance. Infect Agents Dis 4: 115–124. 8548189

[32] Leib DA, Coen DM, Bogard CL, Hicks KA, Yager DR, et al (1989) Immediate-early regulatory gene mutants define different stages in the establishment and reactivation of herpes simplex virus latency. J Virol 63: 759–768. 2536101 10.1128/jvi.63.2.759-768.1989 PMC247748

[33] Hill TJ, Blyth WA, Harbour DA, Berrie EL, Tullo AB (1983) Latency and other consequences of infection of the nervous system with herpes simplex virus. Prog Brain Res 59: 173–184. 6320263 10.1016/S0079-6123(08)63862-5

[34] Sawtell NM (2003) Quantitative analysis of herpes simplex virus reactivation in vivo demonstrates that reactivation in the nervous system is not inhibited at early times postinoculation. J Virol 77: 4127–4138. 12634371 10.1128/JVI.77.7.4127-4138.2003 PMC150616

[35] Neumann DM, Bhattacharjee PS, Hill JM (2007) Sodium butyrate: a chemical inducer of in vivo reactivation of herpes simplex virus type 1 in the ocular mouse model. J Virol 81: 6106–6110. 17360760 10.1128/JVI.00070-07 PMC1900314

[36] Sawtell NM, Thompson RL (1992) Rapid in vivo reactivation of herpes simplex virus in latently infected murine ganglionic neurons after transient hyperthermia. J Virol 66: 2150–2156. 1312625 10.1128/jvi.66.4.2150-2156.1992 PMC289007

[37] Blyth WA, Harbour DA, Hill TJ (1980) Effect of immunosuppression on recurrent herpes simplex in mice. Infect Immun 29: 902–907. 6253401 10.1128/iai.29.3.902-907.1980 PMC551216

[38] Robinson I, Ochsenkuhn MA, Campbell CJ, Giraud G, Hossack WJ, et al (2010) Intracellular imaging of host-pathogen interactions using combined CARS and two-photon fluorescence microscopies. J Biophotonics 3: 138–146. 19670191 10.1002/jbio.200910054

[39] Shaner NC, Campbell RE, Steinbach PA, Giepmans BNG, Palmer AE, et al (2004) Improved monomeric red, orange and yellow fluorescent proteins derived from Discosoma sp. red fluorescent protein. Nature Biotechnology 22: 6. 10.1038/nbt1037 15558047

[40] Foster TP, Kousoulas KG (1999) Genetic analysis of the role of herpes simplex virus type 1 glycoprotein K in infectious virus production and egress. J Virol 73: 8457–8468. 10482598 10.1128/jvi.73.10.8457-8468.1999 PMC112865

[41] Monici M (2005) Cell and tissue autofluorescence research and diagnostic applications. Biotechnol Annu Rev 11: 227–256. 16216779 10.1016/S1387-2656(05)11007-2

[42] Ouyang H, Sun W, Fu Y, Li J, Cheng JX, et al (2010) Compression induces acute demyelination and potassium channel exposure in spinal cord. J Neurotrauma 27: 1109–1120. 20373847 10.1089/neu.2010.1271 PMC2943505

[43] Potel C, Kaelin K, Gautier I, Lebon P, Coppey J, et al (2002) Incorporation of green fluorescent protein into the essential envelope glycoprotein B of herpes simplex virus type 1. J Virol Methods 105: 13–23. 12176138 10.1016/s0166-0934(02)00027-7

[44] Ramachandran S, Knickelbein JE, Ferko C, Hendricks RL, Kinchington PR (2008) Development and pathogenic evaluation of recombinant herpes simplex virus type 1 expressing two fluorescent reporter genes from different lytic promoters. Virology 378: 254–264. 18619637 10.1016/j.virol.2008.05.034 PMC2613845

[45] Snyder A, Bruun B, Browne HM, Johnson DC (2007) A herpes simplex virus gD-YFP fusion glycoprotein is transported separately from viral capsids in neuronal axons. J Virol 81: 8337–8340. 17522199 10.1128/JVI.00520-07 PMC1951318

[46] Antinone SE, Zaichick SV, Smith GA (2010) Resolving the assembly state of herpes simplex virus during axon transport by live-cell imaging. J Virol 84: 13019–13030. 20810730 10.1128/JVI.01296-10 PMC3004300

[47] de Oliveira AP, Glauser DL, Laimbacher AS, Strasser R, Schraner EM, et al (2008) Live visualization of herpes simplex virus type 1 compartment dynamics. J Virol 82: 4974–4990. 18337577 10.1128/JVI.02431-07 PMC2346754

[48] Nagel CH, Dohner K, Fathollahy M, Strive T, Borst EM, et al (2008) Nuclear egress and envelopment of herpes simplex virus capsids analyzed with dual-color fluorescence HSV1(17+). J Virol 82: 3109–3124. 18160444 10.1128/JVI.02124-07 PMC2258981

[49] Tanaka M, Kodaira H, Nishiyama Y, Sata T, Kawaguchi Y (2004) Construction of recombinant herpes simplex virus type I expressing green fluorescent protein without loss of any viral genes. Microbes Infect 6: 485–493. 15109964 10.1016/j.micinf.2004.01.011

[50] Nagel CH, Dohner K, Binz A, Bauerfeind R, Sodeik B (2012) Improper tagging of the non-essential small capsid protein VP26 impairs nuclear capsid egress of herpes simplex virus. PLoS One 7: e44177. 22952920 10.1371/journal.pone.0044177 PMC3432071

[51] Winnard PT Jr, Kluth JB, Raman V (2006) Noninvasive optical tracking of red fluorescent protein-expressing cancer cells in a model of metastatic breast cancer. Neoplasia 8: 796–806. 17032496 10.1593/neo.06304 PMC1715931

[52] von Messling V, Springfeld C, Devaux P, Cattaneo R (2003) A ferret model of canine distemper virus virulence and immunosuppression. J Virol 77: 12579–12591. 14610181 10.1128/JVI.77.23.12579-12591.2003 PMC262577

[53] Griffiths A, Coen DM (2003) High-frequency phenotypic reversion and pathogenicity of an acyclovir-resistant herpes simplex virus mutant. J Virol 77: 2282–2286. 12525666 10.1128/JVI.77.3.2282-2286.2003 PMC140925

[54] Coen DM, Fleming HE Jr, Leslie LK, Retondo MJ (1985) Sensitivity of arabinosyladenine-resistant mutants of herpes simplex virus to other antiviral drugs and mapping of drug hypersensitivity mutations to the DNA polymerase locus. J Virol 53: 477–488. 2982032 10.1128/jvi.53.2.477-488.1985 PMC254660

[55] Coen DM, Kosz-Vnenchak M, Jacobson JG, Leib DA, Bogard CL, et al (1989) Thymidine kinase-negative herpes simplex virus mutants establish latency in mouse trigeminal ganglia but do not reactivate. Proc Natl Acad Sci U S A 86: 4736–4740. 2543985 10.1073/pnas.86.12.4736 PMC287348

[56] Cheng JX, Volkmer A, Book LD, Xie XS (2001) An epi-detected coherent anti-stokes raman scattering (E-CARS) microscope with high spectral resolution and high sensitivity. Journal of Physical Chemistry B 105: 1277–1280.

[57] Cheng JX, Volkmer A, Xie XS (2002) Theoretical and experimental characterization of coherent anti-Stokes Raman scattering microscopy. Journal of the Optical Society of America B-Optical Physics 19: 1363–1375.

[58] Volkmer A, Cheng JX, Xie XS (2001) Vibrational imaging with high sensitivity via epidetected coherent anti-Stokes Raman scattering microscopy. Physical Review Letters 87.

[59] Denk W, Strickler JH, Webb WW (1990) Two-photon laser scanning fluorescence microscopy. Science 248: 73–76. 2321027 10.1126/science.2321027

[60] Helmchen F, Denk W (2005) Deep tissue two-photon microscopy. Nat Methods 2: 932–940. 16299478 10.1038/nmeth818

