# Supplementary figures and images for: Visualization of Mouse Neuronal Ganglia Infected by Herpes Simplex Virus 1 (HSV-1) Using Multimodal Non-Linear Optical Microscopy
Source: PLoS One. 2014 Aug 18;9(8):e105103. doi: 10.1371/journal.pone.0105103 (PMC4136817; doi:10.1371/journal.pone.0105103)

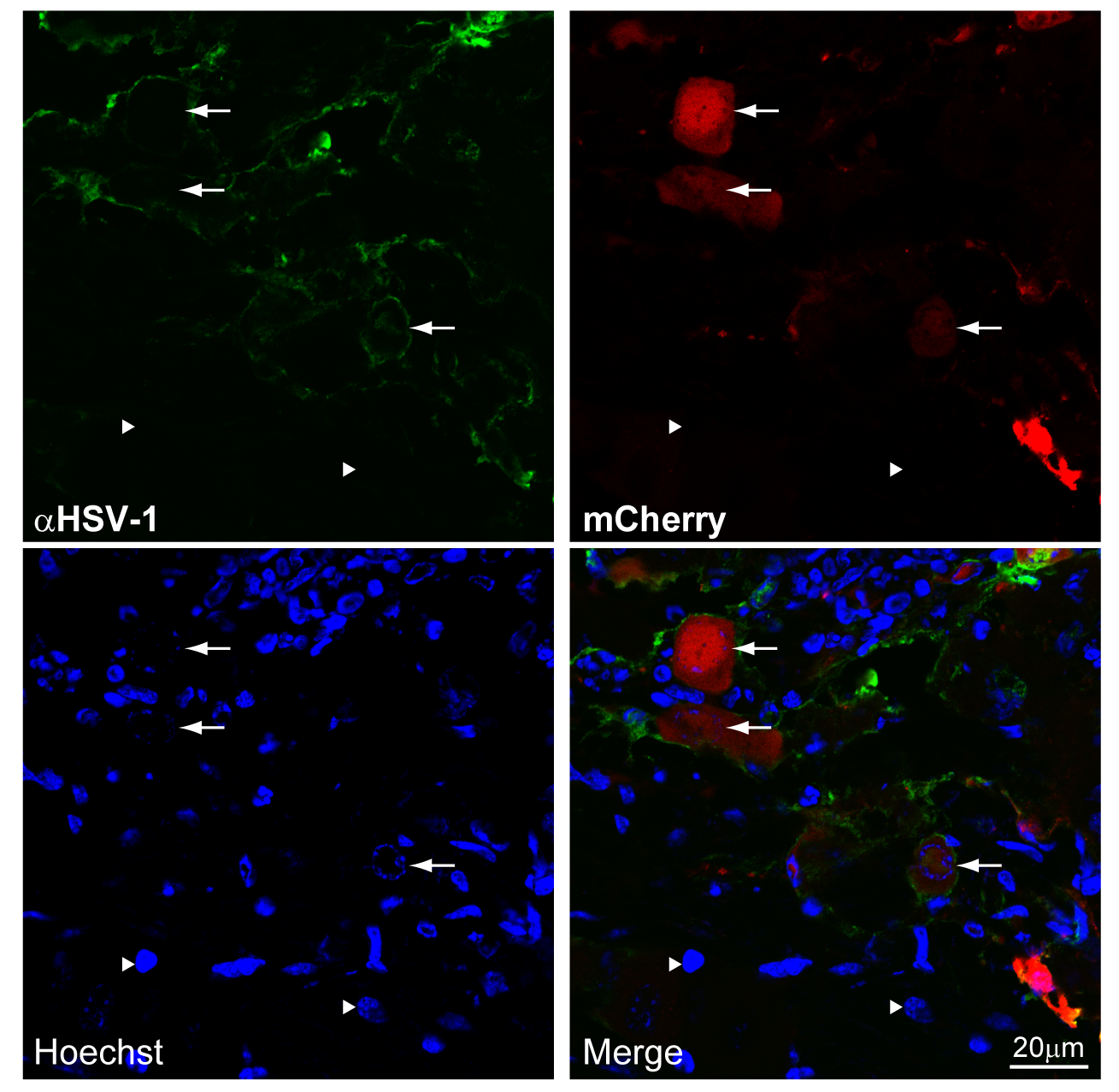

Supplement: Figure S1 — Identification of cells infected with vUs7-8mCherry in TG sections by visualization of mCherry fluorescence and by anti-HSV-1 immunohistofluorescence. TG from mice infected with vUs7-8mCherry were harvested 3 dpi, sectioned, immunostained for HSV-1, and analyzed by confocal microscopy. Shown are infected cells as visualized by immunohistofluorescence using anti-HSV-1 serum (top left panel), and by detection of mCherry fluorescence (top right panel). Nuclei were stained with Hoechst, which labels DNA (bottom left panel). Merged images are shown in the bottom right panel. Arrows point to nuclei of infected cells, arrowheads point to nuclei of non-infected cells. The scale bar represents 20 µm. (TIFF) [file pone.0105103.s001.tiff]
